# Evaluation of possible associated factors for early childhood caries: are preterm birth and birth weight related?

**DOI:** 10.1186/s12903-024-04004-3

**Published:** 2024-02-11

**Authors:** Merve Bilmez Selen, Pınar Demir, Feyza Inceoglu

**Affiliations:** 1 Ankara Tepebaşı Oral and Dental Health Hospital, Ankara, Turkey; 2 Department of Pediatric Dentistry, Faculty of Dentistry, Nuh Naci Yazgan University, Kayseri, Turkey; 3 Department of Medical Statistics, Faculty of Medicine, Malatya Turgut Özal University, Malatya, Turkey

**Keywords:** Birth weight, Infant, Low birth weight, Dental caries, Premature birth, Term birth

## Abstract

**Background:**

This study aimed to evaluate the oral and dental health of preschool children aged 12–71 months living in the Eastern Anatolia Region of Turkey, and to examine the effects of low birth weight (LBW) and preterm, early term and term birth on dental caries.

**Methods:**

475 participants were included in the study. Intraoral examinations were performed and evaluated for the presence of early childhood caries (ECC). These values ​​are; Relationships such as age, gender, birth weight, week of birth, tooth brushing frequency, cariogenic nutrition, and parental education levels were examined. The obtained data were analyzed statistically (chi-square, t-test, artificial neural network (ANN)).

**Results:**

Of the 475 participants, whose parents agreed to fill out the questionnaire, 250 were female and 225 were male. While the mean age was 49.78 ± 14.78 months for those with ECC, it was 38.93 ± 17.96 months for those without. Higher duration of breastfeeding (*p* = 0.04), education level of parents (*p* = 0.001), lower socioeconomic level (*p* = 0.001), and lower brushing frequency (*p* = 0.001) were also found to be significantly associated with ECC. ECC was seen in 90% of 77 children with a history of preterm birth. In LBW, this rate was 83%. According to the ANN result, in preterm birth; 12.9% affected ECC by LBW.

**Conclusion:**

According to the results of our study, both LBW and preterm delivery were found to be associated with ECC and S-ECC (severe early childhood caries). An additional study on parents of preterm/LBW infants would be beneficial. In the early period, regular dental examination, implementation of preventive and preventive treatments, and nutrition education to parents can make a significant difference in the prevention of ECC.

**Supplementary Information:**

The online version contains supplementary material available at 10.1186/s12903-024-04004-3.

## Background

Early childhood caries (ECC) is defined as “the presence of one or more caries (non-cavity or cavity lesion), extracted (due to caries) or filled tooth surfaces in any primary tooth in a child 71 months of age or younger” [1]. Despite the increase in preventive practices to prevent dental caries, ECC remains an important global health problem. If ECC is not treated, it can cause premature loss of primary teeth, malocclusions, speech problems, nutritional deficiencies, serious aesthetic and psychological problems, and adverse effects on the child’s development and quality of life [2].

Tooth decay occurs due to factors including the colonization of complex and infectious bacteria, dietary habits, and oral hygiene. ECC is an infectious disease caused by Streptococcus mutans. For caries formation; cariogenic bacteria, fermentable carbohydrates, and host susceptibility (tooth enamel integrity) are required [3]. In addition to environmental and sociological factors such as the education level and socioeconomic status of the family, developmental disorders such as pre-and postnatal diseases, low birth weight, and preterm birth have also been shown to have effects [4]. Many diseases (nutrition disorders, breathing difficulties, increased tendency to infection, heart disease, chronic liver disorders, eye problems, allergic, physical, mental problems, etc.) can adversely affect the oral-dental health and dental development of the child [2]. Preterm birth occurs before 37 completed weeks of gestation and its global incidence is 11% [5]. In Turkey, the prevalence of preterm birth varies between 10 and 15% in publications from various centers and is reported to be around 12% across Turkey [6, 7]. Low birth weight (LBW) Low birth weight has been defined by WHO as a weight at birth of < 2500 g (5.5 pounds) [8]. The prevalence of LBW is 11% in Turkey [9].

In preterm children, disorders that occur during the amelogenesis of primary and permanent teeth affect enamel formation [10]; It has been suggested that enamel opacities and hypoplasia are the most common dental disorders in premature children [10, 11]. In addition, children with LBW may have a higher risk of developing dental caries due to biological and socioeconomic factors [11].

We aimed to evaluate the oral and dental health, dietary habits, and oral hygiene of preschool children aged 12–71 months living in the Eastern Anatolia Region of Turkey, and to examine the effects of LBW and preterm, early term, and term birth on dental caries.

## Methods

Relevant approvals were obtained from the Non-Invasive Clinical Research Ethics Committee of the Faculty of Medicine of İnönü university (2021/2456) and it was conducted by the principles of the Declaration of Helsinki. The necessary signatures were obtained from the parents of the children who participated in the study with the “Informed Voluntary Consent Form”. The parent had to be literate and agreed to answer the questionnaire.

The study group included children younger than 72 months and their parents who applied to Malatya İnönü University Faculty of Dentistry, Department of Pedodontics between April 2020 and July 2021. The patients have had applied the clinic for respective complaints or oral examinations related with dental caries, orthodontic problems, or dental trauma. The inclusion criteria for the study were children younger than 72 months old with no systemic disease, and the parent had to be literate with no substantial learning difficulty (*n* = 475).

This study’s sample size was determined by power analysis using the G*power 3.1 program. The sample size was determined as 475 with an effect size of 0.20, a margin of error of 0.5, a confidence level of 0.95, and a population representation of 0.95 [12].

The multiple-choice questions were prepared for the survey and evaluated for suitableness. The draft survey was administered to parents of 25 patients who applied to the pedodontics clinic of the university as the pilot study. The completeness of the questions and responses were evaluated, errors were corrected, and the final form was prepared. Twenty-five patients who participated in the pilot study were not included in the study group.

Child’s 24-hour- recall/retrospective food intake, child’s oral and dental health assessment were also recorded. The first three parts of the survey form (socio-demographic and medical evaluation, dietary habits, and oral hygiene habits) were filled in by parents under the supervision of the primary author, for some questions reminding/recall methods were used.

The amount, content, and time of consumption of the food and beverages were specified. To assess the cariogenic score of the nutrients, the pediatric cariogenicity index was used [13]. Each food group’s cariogenicity score was determined from this index. A specialist dietitian was consulted for food group distinction. Cariogenicity scores were calculated separately for solid and liquid foods consumed by each participant. The total cariogenicity score was calculated for each participant using the formula in Evans’ study [13]. A score between 0 and 10 was determined for each participant, and then the mean score was determined by t-test among all.

All the children were examined for dental caries and enamel defects by using a mirror and probe under light and drying the teeth with compressed air. All the evaluations were made by a single pediatric dentist (M.B.S) for standardization purposes. Examinations were conducted in a well-equipped dental clinic with good lighting conditions. Calibration was performed in two sessions, using photographs of clinical cases and extracted primary teeth. There was a 1-week interval between the two calibration sessions, and the intrarater reliability was calculated (k > 0.8). Dental caries were calculated with the decay-missing-filling-tooth(dmft) index [14]. Missing teeth due to trauma and physiological tooth extractions were not included in the dmft score. Conditions that were not considered as caries were as follows: stained or pigmented teeth, discolored occlusal pits or cracks without significant deterioration in enamel structure, dark, shiny, and pitted enamel areas, tooth wear, and erosion. PI and GI were measured using the Silness-Loe plaque index [15]. 3 to 5 years old, 1 or more cavitated, missing (due to caries) or filled flat surface in the milk maxillary anterior teeth, or ≥ 4 (3 years), ≥ 5 (4 years) caries, missing or full score, or ≥ 6 (5 years) age) surfaces were accepted as SECC [1].

Based on the definition of the World Health Organization, children born below 2500 g (5.5 pounds) are “Low birth weight (LBW)”, children born before 37 weeks are “preterm”, and those 37–38 weeks are “early term”, children between 38 and 42 weeks are considered as “term” [8]. Preterm babies were evaluated as extremely (< 28 weeks), very (28–32 weeks), and moderate to late (32–37 weeks) preterm. ECC relationship was investigated according to birth weight (< 2500, ≥ 2500 g) and week (< 37, ≥ 37 weeks) of patients by forming two groups with and without ECC-SECC [11]. In addition, other factors that may affect ECC like sociodemographic information, duration of breastfeeding, nighttime bottle use (months), brush renewal time, solid/liquid food cariogenic score, PI (Plaque Index), and GI (Gingival Index) were also evaluated.

The analysis of the data included in this study was executed with SPSS (Statistical Program in Social Sciences) 26.0 program. The significance level (p) was accepted as 0.05 for comparison tests. Kolmogorov Smirnov test was used to check the conformity of the data to normal distribution. Comparisons in independent pairs; Since the assumption of normality was provided, the significant test (t-test) of the difference between the two means was made. The homogeneity of variance was checked with the Levene test in deciding which test result to use in comparison (*p* > 0.05). In the analysis of categorical data, 2*2 cross tables were created and the phi coefficient(φ) calculated as a result of the chi-square(χ²) test was used in the comparisons. The Cramer coefficient (V) calculated as a result of the chi-square (χ²) test was used in the comparisons made by creating multi-compartment cross tables.

The artificial neural network node (ANN) of the SPSS Modeler program was used and the activation function was allowed to be determined automatically by SPSS Modeler. The sigmoid function was the most preferred activation function in applications [16]. The SPSS program determined the momentum and learning coefficient automatically by iteration method. In SPSS Modeler, ANN (artificial neural network) models, both momentum coefficient and learning coefficient were calculated automatically by iteration.

In the ANN model established in the research, the variables of mother/father education, time to start brushing, frequency of tooth brushing, night breastfeeding, night bottle feeding, PI, GI, cariogenic nutrition score, packaged food consumption, preterm birth, and dentist visits were used. The relationship of these variables with ECC and SECC was evaluated.

## Results

Of the 475 participants who agreed to complete the questionnaire, 250 were girls and 225 were boys. 77% of them had ECC. While the mean age was 49.78 ± 14.78 months for those with ECC, it was 38.93 ± 17.96 months for those without. Among the participants included in the study, it was tested whether the variable groups of gender, mother’s education level, father’s education level, socioeconomic status, duration of breast milk intake, night bottle use, and brush usage frequency affected whether they were ECC or SECC. The results are given in Table 1. Increased duration of breast milk intake (*p* = 0.04), education level of parents (*p* = 0.001), decreased socioeconomic level (*p* = 0.001) and decreased frequency of brushing (*p* = 0.001) were also found to be significantly associated with ECC and SECC (Table 1).

**Table 1:** Evaluation of the relation of ECC between sociodemographic findings, nutrition, and oral hygiene habits

Variable	Group	n / %	ECC		SECC		Total
			(-)	(+)	(-)	(+)	
**Gender**	**Female**	**n**	54_a_	196_a_	83_a_	167_a_	250
		**%**	21.60	78.40	33.20	66.80	100.00
	**Male**	**n**	53_a_	172_a_	72_a_	153_a_	225
		**%**	23.60	76.40	32,00	68.00	100.00
	**p value**		0.610		0.780		
**Mother education status**	**Primary education**	**n**	27_a_	184_b_	48_a_	163_b_	211
		**%**	12.80	87.20	22.70	77.30	100.00
	**High school**	**n**	27_a_	101_a_	41_a_	87_a_	128
		**%**	21.10	78.90	32.00	68.00	100.00
	**License**	**n**	46_a_	77_b_	57_a_	66_b_	123
		**%**	37.40	62.60	46.30	53.70	100.00
	**Postgraduate**	**n**	7_a_	6_b_	9_a_	4_b_	13
		**%**	53.80	46.10	75.00	25.00	100.00
	**p-value**		0.001*		0.001*		
**Father education status**	**Primary education**	**n**	21_a_	141_b_	36_a_	126_b_	162
		**%**	13.00	87.00	22.20	77.80	100.00
	**High school**	**n**	23_a_	114_a_	34_a_	103_b_	137
		**%**	16.80	83.20	24.80	75.20	100.00
	**License**	**n**	55_a_	105_b_	74_a_	86_b_	160
		**%**	34.40	65.60	46.30	53.80	100.00
	**Postgraduate**	**n**	8_a_	8_b_	11_a_	5_b_	16
		**%**	50.00	50.00	68.80	31.30	100.00
	**p-value**		0.001*		0.001*		
**Socioeconomic status**	**Below minimum wage**	**n**	10_a_	64_b_	19_a_	55_a_	74
		**%**	13.50	86.50	25.70	74.30	100.00
	**Minimum wage**	**n**	32_a_	195_b_	52_a_	175_b_	227
		**%**	14.10	85.90	22.90	77.10	100.00
	**Above minimum wage**	**n**	65_a_	109_b_	84_a_	90_b_	174
		**%**	37.40	62.60	48.30	51.70	100.00
	**p value**		0.001*		0.001*		
**Breastfeeding period**	**6 months**	**n**	15_a_	68_a_	30_a_	53_a_	83
		**%**	18.10	81.90	36.10	63.90	100.00
	**12 months**	**n**	23_a_	42_b_	29_a_	36_a_	65
		**%**	35.40	64.60	44.60	55.40	100.00
	**12–24 months**	**n**	50_a_	178_a_	70_a_	158_a_	228
		**%**	21.90	78.10	30.70	69.30	100.00
	**25 months and above**	**n**	12_a_	58_a_	17_a_	53_a_	70
		**%**	17.10	82.90	24.30	75.70	100.00
	**p-value**		0.040*		0.060		
**Nighttime bottle feeding**	**Yes**	**n**	42_a_	166_a_	69_a_	139_a_	208
		**%**	20.20	79.80	33.20	66.80	100.00
	**No**	**n**	65_a_	202_a_	86_a_	181_a_	267
		**%**	24.30	75.70	32.20	67.80	100.00
	**p-value**		0.280		0.820		
**Tooth brushing frequency**	**Two times a day**	**n**	29_a_	34_b_	33_a_	30_b_	63
		**%**	46.00	54.00	52.40	47.60	100.00
	**Once a day**	**n**	37_a_	97_a_	59_a_	75_b_	134
		**%**	27.60	72.40	44.00	56.00	100.00
	**Irregular-none**	**n**	41_a_	237_b_	63_a_	215_b_	278
		**%**	14.70	85.30	22.70	77.30	100.00
	**p-value**		0.001*		0.001*		

n: number, %: percent, ECC; Early childhood caries, S-ECC; Severe early childhood caries.Test value; χ2(chi-square test value), p; statistical significance, *p < 0.05; Different letters in the lines show that there is a difference between the two groups, while the same letters show that there is no difference

It was tested whether all the variables differ between both ECC and SECC statuses. The results are given in Table 2. A statistically significant difference was found between the presence or absence of ECC according to age, birth week, birth weight, toothbrush intake time, solid and liquid food cariogenic score, PI index, and GI index variable values among the participants included in the study (*p* < 0.05; Table 2).

**Table 2:** Comparison of variables in children with and without ECC

Variable		ECC		
		Mean ± SD	Test	p
**Age**	**Absent**	38.93 ± 17.96	-6.351	0.001*
	**Present**	49.78 ± 14.78		
**Birth week**	**Absent**	38.87 ± 1.83	2.618	0.009*
	**Present**	38.12 ± 2.78		
**Birth weight**	**Absent**	3239.21 ± 511.48	2.4	0.017*
	**Present**	3078.92 ± 633.1		
**Breastfeeding (months)**	**Absent**	17.08 ± 8.69	-1.129	0.261
	**Present**	18.28 ± 8.98		
**Nighttime bottle-feeding (months)**	**Absent**	22.79 ± 9.24	-1.478	0.141
	**Present**	25.69 ± 11.86		
**Time to start brushing**	**Absent**	15.91 ± 15.38	-5.642	0.001*
	**Present**	26.46 ± 17.47		
**Solid food cariogenic score**	**Absent**	3.21 ± 0.87	-8.646	0.001*
	**Present**	4.28 ± 1.19		
**Likid food cariogenic score**	**Absent**	1.62 ± 1.02	-5.196	0.001*
	**Present**	2.6 ± 1.87		
**Plaque index (Silness&Löe)**	**Absent**	0.40 ± 0.6	-27.851	0.001*
	**Present**	2.49 ± 0.7		
**Gingival index** (**Silness&Löe)**	**Absent**	0.22 ± 0.48	-23.160	0.001*
	**Present**	2.04 ± 0.77		

ECC; Early childhood caries, SD; standard deviation. Test; test value of significance test of the difference between two means (t), p; statistical significance, *p < 0.05; There is a statistically significant difference between the groups

The relationship between ECC and SECC according to low birth weight (LBW) and term or preterm birth status is given in Table 3. While 16.2% (*n* = 77) of 475 participants had preterm, 4.8% (*n* = 23) had low birth weight. ECC was seen in 90% of 77 children with a history of preterm birth. In low birth weight (LBW), this rate was 83%. The probability of ECC among those born < 37 weeks was significantly higher than among those born ≥ 37 weeks (*p* = 0.003).

**Table 3:** Evaluation of the relationship between LBW, term, preterm birth on ECC-SECC

Variable	Group	n / %	ECC		SECC		Total
			(-)	(+)	(-)	(+)	
**Birth week**	**≥ 37 weeks**	**n /** **%**	99 92.5	299 81.3	140 90.3	258 80.6	398 100.00
	< **37 weeks**	**n /** **%**	8 7.5	69 18.8	15 9.7	62 19.4	77 100.00
**p-value**			0.003*		0.004*		
**Birth history by week**	**preterm 37 weeks>**	**n**	8_a_	69_b_	15_a_	62_b_	77
		**%**	10.40	89.60	19.50	80.50	100.00
	**early term 37–38 weeks**	**n**	18_a_	56_a_	24_a_	50_a_	74
		**%**	24.30	75.70	32.40	67.60	100.00
	**term 38–42 weeks**	**n**	81_a_	243_a_	116_a_	208_b_	324
		**%**	25.00	75.00	35.80	64.20	100.00
**p-value**			0.020*		0.020*		
**Preterm status by week of birth**	**moderate to late preterm (32–37 weeks)**	**n**	0_a_	2_a_	1_a_	1_a_	2
		**%**	11.80	88.20	20.60	79.40	100.00
	**very preterm (28-32-weeks)**	**n**	1_a_	16_a_	1_a_	16_a_	17
		**%**	12.00	88.00	24.00	76.00	100.00
	**extremely preterm (28 weeks>)**	**n**	7_a_	51_a_	13_a_	45_a_	58
		**%**	5.60	94.40	11.10	88.90	100.00
**p-value**			0.738		0.148		
**LBW**	**2500 g >**	**n**	4_a_	19_a_	6_a_	17_a_	23
		**%**	17.40	82.60	26.10	73.90	100.00
**NBW**	**≥ 2500 g**	**n**	103_a_	349_a_	149_a_	303_a_	452
		**%**	22.80	77.20	33.00	67.00	100.00
**p-value**			0.550		0.490		

ECC; Early childhood caries, S-ECC; Severe early childhood caries, LBW; Low birth weight, NBW; normal birth weight. Test value; χ2(chi-square test value), p; statistical significance, *p < 0.05; Different letters in the lines show that there is a difference between the two groups, while the same letters show that there is no difference

The data of the ANN (artificial neural network) model, in which multiple variables are evaluated together, are given in Table 4. Educational status of the mother and father, preterm and LBW, nighttime breastfeeding and sleeping with a bottle, and packaged food consumption were effective on ECC and SECC. The correct prediction rate for ECC and SECC classification was 99.1% and 92% in the training dataset, respectively; in the test data set it was 95.3% and 84.2%. Preterm birth (0.042) and LBW (0,022) were associated with ECC.

**Table 4:** Significance values of factors affecting ECC and SECC

Independent Variable	ECC		SECC	
	P value	Normalized Importance	P value	Normalized Importance
Mother education status	0.042*	***13.0%***	0.039*	***15.3%***
Father education status	0.042*	***13.0%***	0.024*	***9.3%***
Preterm birth history	0.042*	***12.9%***	0.049*	**19.6%**
LBW	0.022*	***6.8%***	0.019*	***7.3%***
The existence of nighttime breast-feeding	0.027*	***8.5%***	0.001*	***0.5%***
The existence of nighttime bottle-feeding	0.011*	***3.4%***	0.019*	***7.4%***
Cariogenic snack intake	0.029*	***9.1%***	0.012*	***4.9%***
First dental visit	0.022*	***6.8%***	0.052	20.6%
Tooth brushing frequency	0.064	19.7%	0.049*	***19.8%***
Time to start brushing	0.065	20.0%	0.066	25.8%
**Plaque index (Silness&Löe)**	0.323	100.0%	0.255	100.0%
**Gingival index** (**Silness&Löe)**	0.169	52.4%	0.202	79.1%
Solid food cariogenic score	0.078	24.3%	0.104	40.9%
Likid food cariogenic score	0.064	19.8%	0.106	41.6%

ECC; Early childhood caries, S-ECC; Severe early childhood caries, LBW;Low birth weight. Test; Artificial neural network (ANN)*p < 0.05; values are statistically significant

## Discussion

Cross-sectional studies are conducted at a single point in time, often in the form of surveys, and for descriptive purposes. In a cross-sectional design, it is possible to record exposure to many risk factors and evaluate multiple outcomes. Gender, educational status of the mother and father, socioeconomic status, prevalence of nighttime breastfeeding, LBW and preterm birth were identified as risk variables for both ECC and S-ECC in the ANN model in the present study. It has been shown that the education level of the parents is related to the presence and severity of ECC in children, and low caries prevalence and mean dmft values have been recorded in the children of families with a high education level [17, 18]. In our study, it was observed that the prevalence of caries in children decreased as the education level of the parents increased the study of Özen et al., it was found that brushing before 18 months had a significant effect on preventing ECC [19]. Özen et al. [18] found that brushing teeth before 18 months reduces the occurrence of ECC. In the present study, the first toothbrush purchasing time has been found to be late in both ECC and S-ECC groups. PI and GI scores also verify this data. When the studies conducted on preschool children in Turkey were evaluated, it was observed that the average dmft value was between 2 and 8 [19, 20]. It was observed in this study that the incidence of tooth decay in preschool children was similar to previous years. Although some researchers reported that boys have a 38% higher risk of developing ECC than girls, in this study, no difference was found between the genders [21]. It is stated that the prevalence of caries increases due to the increase in the number of affected teeth with increasing age and the prolongation of the exposure time of the teeth to the cariogenic environment [4, 22]. The results of this study also support this finding. The average age of children with ECC was around 4.5 years, while that of children without ECC was around 3.5 years.

Özer et al. found a significant relationship between ECC prevalence and bottle feeding while sleeping (*p* < 0.05) [20]. According to the ANN data of this study, nighttime bottle use and nighttime breast milk intake were found to affect ECC and SECC (*p* < 0.05). Solid and liquid food cariogenicity scores were also significant for both ECC and SECC in this study (*p* = 0.001). In Evans’ study, however, there was no statistically significant difference between the groups’ mean solid food cariogenicity scores. The fluid cariogenicity score was higher in the SECC group compared to the children without caries [13]. In this study, unlike Evans, the solid cariogenic nutrition score was found to be higher than the fluid score in both the ECC group and the control group. Consuming more acidic beverages in the USA and consuming probiotic-containing beverages such as ayran in Turkey may have led to this result.

While it has been reported that preterm children have more carious tooth surfaces than term children, there are also studies indicating that there is no relationship [4, 23, 24]. In the ANN model established in this study, it was observed that the risk of ECC and SECC decreased as the week of birth increased, and the difference was found to be significant (*p* = 0.02). Preterm birth affected ECC by 12.9% and SECC by 19.6%. The low surface quality of the primary tooth enamel and the thin enamel thickness in these teeth increase the susceptibility of teeth to caries attacks [10, 25]. The relationship between ECC and SECC was not found to be significant according to the late, moderate, and severe preterm status of those with a history of preterm birth. Twetman et al. reported that babies born mid to late preterm and babies with LBW are more likely to have early childhood caries at the age of 5 years [5]. Saraiva et al. also reported that preterm birth is associated with dental caries [26]. In the literature, higher dmft values have been recorded in children with LBW [17]. In a study in which the relationship between LBW and ECC was observed in children aged 2–5 years, it was stated that the relationship between LBW-ECC became statistically significant when 2-year-old children were excluded from the study. This is explained by the fact that the teeth in 2-year-old children do not have enough time for caries formation due to newly erupted or still erupting teeth [26]. In this study, there was a relationship between LBW and both ECC and SECC according to the ANN model (*p* = 0.022, *p* = 0.019). LBW; was found to affect ECC by 6.8% and SECC by 7.3%.

## Conclusions

According to the results of our study, both LBW and preterm birth were associated with ECC and SECC. Instinctively feeding these children with foods high in carbohydrates to accelerate their growth may also be an important factor in the formation of ECC. Therefore, for preterm and LBW children, regular dental examinations, implementation of preventive treatments, oral hygiene, and nutrition education for parents can make a significant difference in the prevention of ECC. Informing public health advocates, obstetricians, pediatricians, neonatal nurses, and family physicians on this issue to provide early intervention is extremely important.

The limitation of this study is that it was conducted on a sample of parents with a deficient education and socioeconomic level. Since there is a population of parents who have difficulty understanding the importance of milk teeth in the region where the study was conducted and prefer extraction instead of treatment for economic reasons, preventive applications and early interventions are impossible. In addition, parents’ neglect of dental visits made it impossible to diagnose enamel hypoplasia and white spots, which are common in preterm and LBW children. Because these formations became cavitated caries when oral hygiene deficiency was added. Therefore, a susceptibility factor that would normally have little effect may have had more severe consequences in this population.

### Electronic supplementary material

Below is the link to the electronic supplementary material.

Supplementary Material 1

Supplementary Material 2

## Data Availability

All data generated or analyzed during this study are included in this published article [and its supplementary information files]. The excel data sets used and/or surveys analyzed during the current study are available from the corresponding author upon reasonable request. We guarantee that the data will be shared if requested by your journal.

## Abbreviations

ECC: Early childhood caries
LBW: Low birth weight
NBW: Normal birth weight
dmft: Decay-missing-filling
ANN: Artificial nöral network
PI: Plaque index
GI: Gingival index
WHO: World Health Organization
SPSS: Statistical Program in Social Sciences
