# Supplementary material for: Evaluation of possible associated factors for early childhood caries: are preterm birth and birth weight related?
Source: BMC Oral Health. 2024 Feb 11;24:218. doi: 10.1186/s12903-024-04004-3 (PMC10859008; doi:10.1186/s12903-024-04004-3)
Supplement: Supplementary file 1 — Supplementary Material 1 [file 12903_2024_4004_MOESM1_ESM.docx]

**Title:** Evaluation of The Effect of Preterm Birth and LBW on ECC: A cross-sectional study

**Short Title:** Dental caries and premature birth relationship

**1.**MERVE BİLMEZ SELEN (Corresponding author) collected the data and led the writing.

e.mail: [mervebilmez89@gmail.com](mailto:mervebilmez89@gmail.com)

GSM: 05457673089

Orcid ID: [0000-0003-0726-4992](https://orcid.org/0000-0003-0726-4992)

Affiliation: Ankara Tepebaşı Oral and Dental Health Education and Research Hospital, Ankara, Turkey

**2.** PINAR DEMİR conceived the ideas and led the writing.

e.mail: [pinardemir101@hotmail.com](mailto:pinardemir101@hotmail.com)

GSM: 05331431656

Orcid ID: 0000-0003-2030-5429

Affiliation: Department of Pediatric Dentistry, Faculty of Dentistry, Nuh Naci Yazgan University, Kayseri, Turkey

**3.** FEYZA İNCEOĞLU analyzed the data.

e.mail: [feyza.inceoglu@ozal.edu.tr](mailto:feyza.inceoglu@ozal.edu.tr)

GSM: 05069316595

Orcid ID: 0000-0003-1453-0937

Affiliation: Department of Medical Statistics, Turgut Özal University, Faculty of Medicine, Malatya, Turkey

**Word count:** 2750 (excluding abstract, tables, and references)

**Conflicts of interest/Competing interests:** Author Merve Bilmez Selen, Pinar Demir, and Feyza Inceoğlu declare that they have no conflict of interest.

**Availability of data and material:** The data sets used and/or analyzed during the current study are available from the corresponding author upon reasonable request. We guarantee that the data will be shared if requested by your journal.
