# Supplementary material for: Evaluation of possible associated factors for early childhood caries: are preterm birth and birth weight related?
Source: BMC Oral Health. 2024 Feb 11;24:218. doi: 10.1186/s12903-024-04004-3 (PMC10859008; doi:10.1186/s12903-024-04004-3)
Supplement: Supplementary file 2 — Supplementary Material 2 [file 12903_2024_4004_MOESM2_ESM.docx]

Dear Editor,

Thank you very much for considering our article. We added reviewer comments to the "point-by-point responseto reviewers" file. We made the requested changes. We crossed out the parts we removed. We wrote the parts we changed in red. We are looking forward to your valuable comments.

Kind regards.
